# Bioinformatic Identification and Experimental Validation of Novel Antioxidant Peptides Derived from Salmon Collagen

**DOI:** 10.3390/foods15162882

**Published:** 2026-08-18

**Authors:** Suleivys M. Nuñez, Camila B. Barrera, Ignacio S. del Río, Jaime E. Gómez López, Tanya Roman, Fanny Guzmán, Constanza Cárdenas

**Affiliations:** 1 Escuela de Ingeniería Química, Pontificia Universidad Católica de Valparaíso, Valparaíso 2340025, Chile; camila.barrera.m@mail.pucv.cl (C.B.B.); ignacio.delrio.p@mail.pucv.cl (I.S.d.R.); jaime.gomez@pucv.cl (J.E.G.L.); 2 Programa de Doctorado en Biotecnología, Pontificia Universidad Católica de Valparaíso/Universidad Técnica Federico Santa María, Valparaíso 2340025, Chile; tanya.roman.b@mail.pucv.cl; 3 Núcleo de Biotecnología Curauma (NBC), Pontificia Universidad Católica de Valparaíso, Valparaíso 2373223, Chile; fanny.guzman@pucv.cl; 4 Centro de Investigación Interdisciplinaria en Biomedicina, Biotecnología y Bienestar (CID3B), Pontificia Universidad Católica de Valparaíso, Valparaíso 2340000, Chile

**Keywords:** bioactive peptides, in silico screening, peptide synthesis, cellular antioxidant activity, fish-processing by-products, functional ingredients

## Abstract

The objective of this study was to identify and experimentally validate novel antioxidant peptides derived from salmon collagen through an integrated bioinformatic and experimental workflow. Collagen sequences from *Salmo salar* were subjected to in silico enzymatic hydrolysis, and the resulting peptides were characterized using physicochemical descriptors. Principal component analysis and k-means clustering were then applied as exploratory tools to prioritize candidates displaying physicochemical proximity to previously reported antioxidant peptides. The selected candidates were chemically synthesized, purified, and evaluated at concentrations of 0.2 and 0.4 mM using the 2,2-diphenyl-1-picrylhydrazyl (DPPH) radical-scavenging assay, the 2,2′-azino-bis(3-ethylbenzothiazoline-6-sulfonic acid) (ABTS) radical-scavenging assay, and a cellular antioxidant activity assay, with vitamin C as a positive control. Cytotoxicity was assessed using the WST-1 assay. The peptide AS-5390 (DGCERCF) demonstrated superior radical scavenging capacity, with activity comparable to that of vitamin C in the DPPH assay at 0.2 mM and maintaining antioxidant activity in the ABTS assay at both tested concentrations. The peptide AS-5394 (PGDIG) exhibited the strongest cellular antioxidant response, reaching approximately 40% activity at 0.4 mM. Both peptides maintained high cell viability. Overall, this integrated strategy enabled the identification of salmon collagen-derived peptides with assay-dependent antioxidant activity and potential applications as functional food or nutraceutical ingredients.

## 1. Introduction

Oxidative processes are key drivers of food deterioration and oxidative stress-related disorders in humans. In food systems, lipid and protein oxidation reduce shelf life, sensory quality, and nutritional value, whereas in biological systems, excessive production of reactive oxygen species (ROS) is associated with chronic diseases, aging, and cellular dysfunction [1,2]. This dual relevance has intensified the search for antioxidants that are not only effective but also safe, sustainable, and compatible with food and health-related applications.

Bioactive peptides derived from food proteins have attracted increasing attention as natural antioxidants due to their multifunctionality, bioavailability, and structural diversity [3,4]. Their activity may involve radical scavenging, metal ion chelation, and modulation of cellular redox responses, depending on amino acid composition, sequence, molecular weight, charge, hydrophobicity, and other physicochemical properties [5,6,7]. Therefore, identifying sequence-level structure–activity relationships is essential for developing peptide-based functional ingredients with reproducible antioxidant performance.

Collagen is a major structural protein of the extracellular matrix and a principal component of connective tissues in animals, including skin, bones, cartilage, and tendons, where it provides mechanical strength and structural support [8]. Among available protein sources, marine collagen is a promising substrate for discovering antioxidant peptides. Collagen-rich by-products from fish processing are abundant, underutilized, and relevant to circular economy strategies [8,9]. Several studies have shown that collagen hydrolysates and peptide fractions from fish skin, scales, and bones exhibit antioxidant, techno-functional, and biological properties [10,11,12]. Salmon collagen is particularly relevant because the salmon industry generates large volumes of by-products rich in collagen. At the same time, collagen-derived sequences are rich in residues such as glycine, proline, hydroxyproline, and sulfur-containing amino acids that may contribute to antioxidant activity [6].

Despite the growing evidence supporting the antioxidant potential of marine collagen-derived peptides, the current literature remains focused primarily on complex hydrolysates, peptide mixtures, or molecular-weight fractions rather than on individual, well-defined peptide sequences. This limits reproducibility, hinders mechanistic interpretation, and makes it difficult to connect specific structural features with antioxidant function. Conventional workflows involving protein hydrolysis, fractionation, purification, activity-guided screening, and peptide identification remain essential for the experimental discovery and validation of bioactive peptides. However, these procedures can be labor-intensive and time-consuming, particularly when large numbers of potential peptide sequences must be evaluated. Bioinformatics-assisted approaches can complement these experimental workflows by facilitating candidate prioritization, reducing the number of sequences requiring synthesis and experimental testing, and making the overall discovery process more targeted and practical [13,14,15,16].

Computational digestion, physicochemical descriptor analysis, dimensionality reduction, clustering, and activity prediction can be used to screen large peptide libraries before synthesis, thereby reducing the experimental search space while preserving the need for subsequent experimental validation [15,16]. Integrated in vitro and in silico strategies have also been reported for the discovery of antioxidant peptides from various food-protein sources, including sunflower, flixweed, camelina, and broken-rice proteins. These studies combined peptide prediction or identification with chemical antioxidant assays and, in some cases, cellular models [17,18,19]. Although they provide relevant methodological precedents, they differ from the present study in the protein source and in the specific sequence of candidate prioritization and experimental validation. The present work extends these integrated approaches to salmon collagen by combining in silico enzymatic hydrolysis, physicochemical descriptor analysis, principal component analysis, and clustering to prioritize candidates before chemical synthesis.

Another limitation in the field is the frequent reliance on a single chemical antioxidant assay. Chemical antioxidant capacity can be evaluated using complementary methods based on different reaction mechanisms. Representative electron-transfer-based approaches include the ferric reducing antioxidant power (FRAP) and cupric ion reducing antioxidant capacity (CUPRAC) assays, whereas the oxygen radical absorbance capacity (ORAC) assay is primarily based on hydrogen atom transfer. Radical-scavenging assays such as 2,2-diphenyl-1-picrylhydrazyl (DPPH) and 2,2′-azino-bis(3-ethylbenzothiazoline-6-sulfonic acid) (ABTS) are also widely used and may involve electron- and/or hydrogen-transfer processes depending on the experimental conditions [7,20,21]. Because each chemical assay captures only a specific aspect of antioxidant behavior in simplified systems, no single method provides a complete assessment. Moreover, these assays do not fully account for peptide stability, cellular interactions, intracellular availability, or modulation of oxidative stress in biological environments. Therefore, combining complementary chemical assays with cell-based models provides a more comprehensive and biologically informative evaluation of antioxidant potential [7,20,22,23].

Based on this gap, the novelty of the present study lies in the integrated bioinformatic–experimental identification of novel antioxidant peptides derived specifically from salmon collagen, progressing from in silico peptide generation to sequence-level synthesis and multi-assay validation. Collagen sequences from *Salmo salar* were subjected to in silico enzymatic hydrolysis, and the resulting peptide library was screened using physicochemical descriptors, principal component analysis, and clustering to prioritize candidates with antioxidant potential. The selected peptides were chemically synthesized and evaluated using complementary chemical and cellular antioxidant activity assays, followed by cytotoxicity testing of the most promising candidates. By linking computational prioritization with experimental confirmation at the sequence level, this study provides new evidence on structure–activity relationships of salmon collagen-derived peptides. It supports their potential development as sustainable functional ingredients for food, nutraceutical, and health-related applications.

## 2. Materials and Methods

### 2.1. In Silico Prediction of Peptide Sequences with Antioxidant Activity

Antioxidant peptides were predicted using the following in silico pipeline:

First, a targeted, structured bibliographic search was conducted for candidate reference selection using WoS, Scopus, ScienceDirect, and Google Scholar to identify previously reported antioxidant peptide sequences from different biological and food-protein sources. The search terms included “antioxidant peptide,” “antioxidant bioactive peptide,” “peptide sequence,” “radical scavenging peptide,” and “cellular antioxidant activity peptide.” The search aimed to collect experimentally evaluated antioxidant peptides and obtain the information required for their subsequent use as reference sequences in the bioinformatic analysis. Relevant publications were reviewed, with preference given to recent studies (2013–2023) reporting individual peptide sequences and their antioxidant properties. From each selected study, the complete amino acid sequence, biological source, antioxidant assay used, and available physicochemical information were extracted. The final dataset consisted of 23 peptide sequences that contained sufficient information for calculating and comparing physicochemical descriptors with peptides generated from salmon collagen. This dataset was not intended to represent a systematic or exhaustive compilation of all antioxidant peptides reported in the literature, but rather a selected reference panel for exploratory multivariate analysis and candidate prioritization (Table 1).

Second, collagen protein sequences from *Salmo salar* were retrieved from the NCBI Protein database as RefSeq records (Table 2). Available sequences representing different collagen types were included to broaden the sequence diversity evaluated in the computational analysis. Only sequences longer than 1000 amino acids were retained as an operational selection criterion to prioritize long collagen protein sequences and provide extensive sequence coverage for subsequent in silico enzymatic hydrolysis. Thus, sequence selection was based on collagen-type diversity, sequence length, and availability in NCBI, rather than on the experimentally determined abundance of individual collagen types in a specific salmon-processing by-product. These protein sequences were used exclusively as computational substrates for peptide prediction. Accordingly, the resulting peptides were predicted from salmon collagen sequences and were not identified directly in an experimentally produced salmon collagen hydrolysate.

Third, in silico enzymatic hydrolysis was performed using the BIOPEP platform [36]. Subtilisin (EC 3.4.21.62) was selected among the proteases available in BIOPEP to represent a potentially scalable industrial protein-hydrolysis process rather than gastrointestinal digestion. Subtilisin is a serine endopeptidase with broad specificity for peptide bonds, and subtilisin-type commercial proteases have been widely applied to the production of bioactive peptides from food proteins and food-industry by-products [37]. A single enzyme was intentionally considered to generate a defined and reproducible peptide library for subsequent physicochemical and multivariate analyses. Because protease specificity directly determines the cleavage pattern and the resulting peptide sequences, the predicted library is specific to subtilisin hydrolysis and would differ from libraries generated using digestive or alternative industrial proteases. The resulting peptide fragments were filtered to retain unique sequences containing at least five amino acids. This operational cut-off was selected to preserve sufficient sequence complexity for physicochemical descriptor calculation and multivariate discrimination, while maintaining a manageable peptide library for subsequent candidate selection, chemical synthesis, and experimental characterization. The threshold was not intended to indicate that shorter peptides lack antioxidant activity, but rather to define the scope of the present screening workflow.

Fourth, clustering and similarity analyses were conducted using the Peptides package [38] in RStudio (V 2026.01.2-418). A total of 15 selected physicochemical descriptors (Supplementary Table S1) were calculated for a dataset comprising reference peptides (Table 1) and unique fragments obtained from in silico hydrolysis (Supplementary Table S2). Data were normalized and subjected to principal component analysis (PCA), followed by k-means clustering [39]. Two complementary clustering resolutions were examined: k = 20, which provided a finer partition of the physicochemical space, and k = 11, which provided a broader and more parsimonious grouping. Comparison of both solutions was used to evaluate whether the physicochemical proximity between collagen-derived candidates and the reference peptide panel was maintained under different cluster resolutions. Neither solution was considered a confirmatory classifier of antioxidant activity.

Finally, candidates were prioritized through the combined interpretation of their PCA distribution, physicochemical descriptor profiles, and cluster membership under both k = 20 and k = 11. Physicochemical proximity to the reference peptide panel was considered only as an exploratory screening criterion. Sequences with highly redundant amino acid compositions or those considered difficult to synthesize by solid-phase peptide synthesis were subsequently excluded. Therefore, clustering supported candidate prioritization but did not establish antioxidant activity, which required experimental validation. 

### 2.2. Experimental Validation of the Obtained Peptide Sequences

#### 2.2.1. Chemical Synthesis and Characterization of the Selected Peptides

The peptide set selected for synthesis comprised the in silico-predicted peptides obtained in Section 2.1 and three peptides used as references for activity: VIAPW [24,25], TSSSLNMAVRGGLTR [27], and VAAGRTDAGVH [26].

The peptides were synthesized using solid-phase peptide synthesis (SPPS) on Rink amide resin, employing the Fmoc/tBu strategy by simultaneous synthesis in “tea bags” [40]. Briefly, one “tea bag” with 40 mg of Rink amide resin was used for each peptide. Deprotection steps to remove the Fmoc protecting group were performed using 20% piperidine in dimethylformamide (DMF). Coupling reactions were carried out with solutions containing the amino acid, an activator (HBTU or HCTU), OxymaPure, and DIPEA, using DMF as the main solvent. After chain assembly was completed, peptide cleavage was performed with a solution of 92.5% trifluoroacetic acid (TFA), 2.5% triisopropylsilane (TIS), 2.5% water, and 2.5% 2,2′-(ethylenedioxy)diethanethiol for peptides containing cysteine, methionine, or tryptophan, and with 95% TFA, 2.5% TIS, and 2.5% water for the remaining peptides. The peptides were then precipitated in cold ether.

Purification was performed using Sep-Pak C18 columns with an acetonitrile–water gradient ranging from 0 to 100%. The purified aqueous peptide solutions were frozen in liquid nitrogen and lyophilized for use.

The identity of the synthesized peptides was confirmed by electrospray ionization mass spectrometry (ESI-MS) (Shimadzu Corporation, Kyoto, Japan), and purity was assessed by reverse-phase high-performance liquid chromatography (RP-HPLC) (JASCO Corporation, Tokyo, Japan) using a water–acetonitrile gradient from 0 to 70% [40]. All peptides showed purities higher than 90%.

#### 2.2.2. Antioxidant Activity with the DPPH Scavenging Assay

The antioxidant activity of the peptides was evaluated at two preliminary screening concentrations of 0.2 and 0.4 mM, selected for comparative assessment rather than to establish complete concentration–response curves. Vitamin C was used as a positive control in all assays, and the DPPH radical-scavenging method was performed according to [4]. For each assay, 140 µL of sample was mixed with 1.4 mL of DPPH solution (30 mg/L). The blank contained the same ratio of sample to water, while the control contained DPPH and water. The mixtures were incubated in the dark at room temperature for 1 h with constant stirring, and the absorbance was subsequently measured at 520 nm. The radical scavenging capacity was calculated as:

(1) $$Scavenging\;effect\;\left(\%\right)=\left[\frac{{{Abs}_{control\;}\;-\;(Abs}_{sample\;}-\;{Abs}_{blank\;})}{{Abs}_{control\;}}\right]\cdot 100,$$

where *Abs_sample_* is the absorbance of peptides with DPPH, *Abs_control_* is the absorbance of DPPH without peptides, and *Abs_blank_* is the absorbance of peptides without DPPH.

#### 2.2.3. Antioxidant Activity with the ABTS Scavenging Assay

The same preliminary screening concentrations of 0.2 and 0.4 mM were evaluated using the ABTS radical-scavenging method [4], with vitamin C as a positive control. The ABTS radical scavenging solution was prepared by mixing 7 mM ABTS with 2.45 mM potassium persulfate in distilled water (1:1), followed by incubation for 12 h. The resulting solution was diluted 50-fold in 99.99% ethanol to obtain an absorbance of approximately 0.7. For each tube, 15 µL of sample was mixed with 1.5 mL of the diluted ABTS solution. For the blank, the peptide solution was replaced with ethanol while maintaining the same proportions. Samples were incubated in the dark at room temperature for 6 min with constant shaking, and absorbance was measured at 734 nm. No Trolox calibration curve was performed; therefore, the results were expressed as ABTS inhibition (%) rather than Trolox equivalent antioxidant capacity (TEAC). ABTS inhibition was calculated as:

(2) $$ABTS\;inhibition\;\left(\%\right)=\frac{\left(A_{0}\;-{\;A}_{m}\right)}{A_{0}}\cdot 100,$$

where *A_m_* is the absorbance of the sample, and *A*_0_ is the absorbance of the blank.

#### 2.2.4. Cellular Antioxidant Activity (CAA)

The CAA was assessed by measuring ROS-associated intracellular fluorescence [22] using the RTgill-W1 cell line (ATCC CRL-2523). Cells were cultured in Leibovitz’s L-15 medium supplemented with 10% fetal bovine serum and maintained at 18 °C. Once the required confluence was reached, adherent cells were washed with phosphate-buffered saline (PBS) and detached by trypsinization. Cell concentration and viability were determined using a Neubauer counting chamber and Trypan Blue exclusion. Viable cells were subsequently resuspended in complete L-15 medium. Cells were seeded at a density of 600,000 cells/well in 96-well plates and incubated for 48 h at 18 °C. Lyophilized peptides were resuspended directly in L-15 medium immediately before treatment. After incubation, the culture medium was removed, and 90 µL of DCFH-DA solution (33.33 µM) and 10 µL of peptide solution were added to each well to obtain final peptide concentrations of 0.2 and 0.4 mM. The plates were incubated for 1 h at 18 °C. The treatment solution was then removed, and 100 µL of PBS was added to each well. Controls were defined as: blank (cells only), negative control (cells + DCFH-DA), and positive control (cells + DCFH-DA + 100 µL of AAPH as a ROS inducer). Fluorescence was measured every minute for 60 min at excitation and emission wavelengths of 485 and 535 nm, respectively, using a VersaMax microplate reader (Molecular Devices, LLC, San Jose, CA, USA).

Fluorescence–time curves were integrated using GraphPad Prism (V 11.0.2) to calculate the area under the curve (AUC). The CAA was calculated as:

(3) $$CAA\;(\%)=\frac{{AUC}_{N}\;-\;{AUC}_{S}}{{AUC}_{N}}\cdot 100,$$

where *AUC*_*N*_ is the area under the fluorescence–time curve of the negative control, and *AUC*_*S*_ is the area under the fluorescence–time curve of the synthetic antioxidant peptides. Each treatment and control was evaluated in three technical replicate wells.

### 2.3. In Vitro Cytotoxicity Using WST-1 Assay

Cytotoxicity was experimentally evaluated using the WST-1 cell proliferation assay [41] with the RTgill-W1 cell line (ATCC CRL-2523). Cell culture, washing, trypsinization, cell counting, viability assessment by Trypan Blue exclusion, and peptide resuspension in L-15 medium were performed following the same procedure described in Section 2.2.4.

The viable-cell suspension was adjusted to 7.0 × 10^4^ cells/mL in complete L-15 medium, and 100 µL was seeded per well in 96-well plates. Cells were incubated for 24 h at 18 °C until approximately 80% confluence, and the formation of a cell monolayer was reached. Subsequently, 10 µL of each peptide solution was added to obtain final concentrations of 0.4 and 0.8 mM. Each peptide concentration and the corresponding controls were evaluated in three technical replicate wells. Cells were exposed to the peptides for 48 h at 18 °C without agitation.

After the exposure period, 10 µL of WST-1 reagent was added to each well, and the plates were incubated for an additional 4 h at 18 °C. Untreated cells were used as the negative control, whereas 10 µL of Triton X-100 was added to each well to obtain a final concentration of 2% (*v*/*v*), serving as the positive cytotoxicity control. Absorbance was measured at 450 nm using a VersaMax microplate reader (Molecular Devices, LLC., San Jose, CA, USA). Cell viability was calculated as:

(4) $$Cell\;viability\;(\%)=\frac{{Abs}_{sample}}{{Abs}_{control}}\cdot 100,$$

where *Abs_sample_* is the absorbance of peptide-treated cells, and *Abs_control_* is the absorbance of untreated cells.

### 2.4. Statistical and Multivariate Analyses

The data are expressed as mean ± standard deviation from three technical replicates. Each assay (DPPH, ABTS, CAA, and WST-1) was analyzed separately, and all peptide treatments at all evaluated concentrations were compared simultaneously within the corresponding assay. Each peptide–concentration combination was treated as a distinct treatment group. Before statistical analysis, the normality of the model residuals and the homogeneity of variances were assessed using the Shapiro–Wilk and Levene tests, respectively. The assumptions were considered satisfied when *p* > 0.05. For the DPPH, ABTS, and CAA assays, the assumptions of residual normality and homogeneity of variances were not satisfied (*p* ≤ 0.05). Therefore, differences among peptide–concentration groups were evaluated using the Kruskal–Wallis test. When the global Kruskal–Wallis test was significant, pairwise comparisons of mean ranks were performed using the multiple-comparison procedure implemented in InfoStat. For the WST-1 assay, both assumptions were satisfied (*p* > 0.05); therefore, differences among treatments were evaluated using one-way analysis of variance (ANOVA). When the global ANOVA was significant, multiple comparisons among treatment means were performed using Fisher’s least significant difference (LSD) test. A *p*-value < 0.05 was considered statistically significant. Because different statistical procedures were applied, significance letters were displayed only in the WST-1 figure. To avoid visual overcrowding caused by the large number of peptide–concentration groups and associated significance letters, pairwise-comparison letters were not displayed in the DPPH, ABTS, and CAA figures. All statistical analyses were performed using InfoStat/L (V 2020) software.

For the in silico prediction of peptide candidates, multivariate analyses were performed in RStudio. A matrix comprising 15 physicochemical descriptors calculated for the 597 unique salmon collagen-derived peptides and the 23 reference antioxidant peptides was normalized prior to analysis. Principal component analysis (PCA) was applied to reduce the dimensionality of the dataset, and the first five principal components, which together explained 89% of the total variance, were retained for subsequent analysis. K-means clustering was then applied to these components using two complementary resolutions, k = 20 and k = 11. The k = 20 solution was examined to provide finer discrimination among physicochemical profiles, whereas k = 11 was used to obtain a broader grouping and assess the consistency of candidate–reference associations at a lower cluster resolution. Both solutions were considered jointly during candidate prioritization. These multivariate analyses were exploratory and were used to identify physicochemical similarities between collagen-derived peptides and previously reported antioxidant peptides; they were not used for inferential hypothesis testing or as evidence confirming antioxidant activity. Physicochemical descriptors were calculated using the Peptides package, and the analyses and graphical representations were performed in RStudio.

## 3. Results and Discussion

### 3.1. In Silico Prediction of Peptide Sequences with Antioxidant Activity

In silico enzymatic hydrolysis of the selected NCBI RefSeq salmon collagen protein sequences using the BIOPEP platform generated approximately 14,000 predicted peptide fragments. These sequences represent computationally generated candidates and were not identified in an experimentally produced salmon collagen hydrolysate. After applying a minimum-length criterion of at least five amino acids and removing duplicate sequences, a dataset of 597 unique predicted peptides was obtained (Supplementary Table S2). The resulting dataset was complemented with a selected panel of 23 experimentally evaluated antioxidant peptide sequences used as physicochemical references for exploratory multivariate analysis and candidate prioritization (Table 1). Physicochemical descriptors were calculated and subjected to principal component analysis (PCA), which explained 89% of the total variance with five components (Supplementary Table S3).

**Figure 1:**
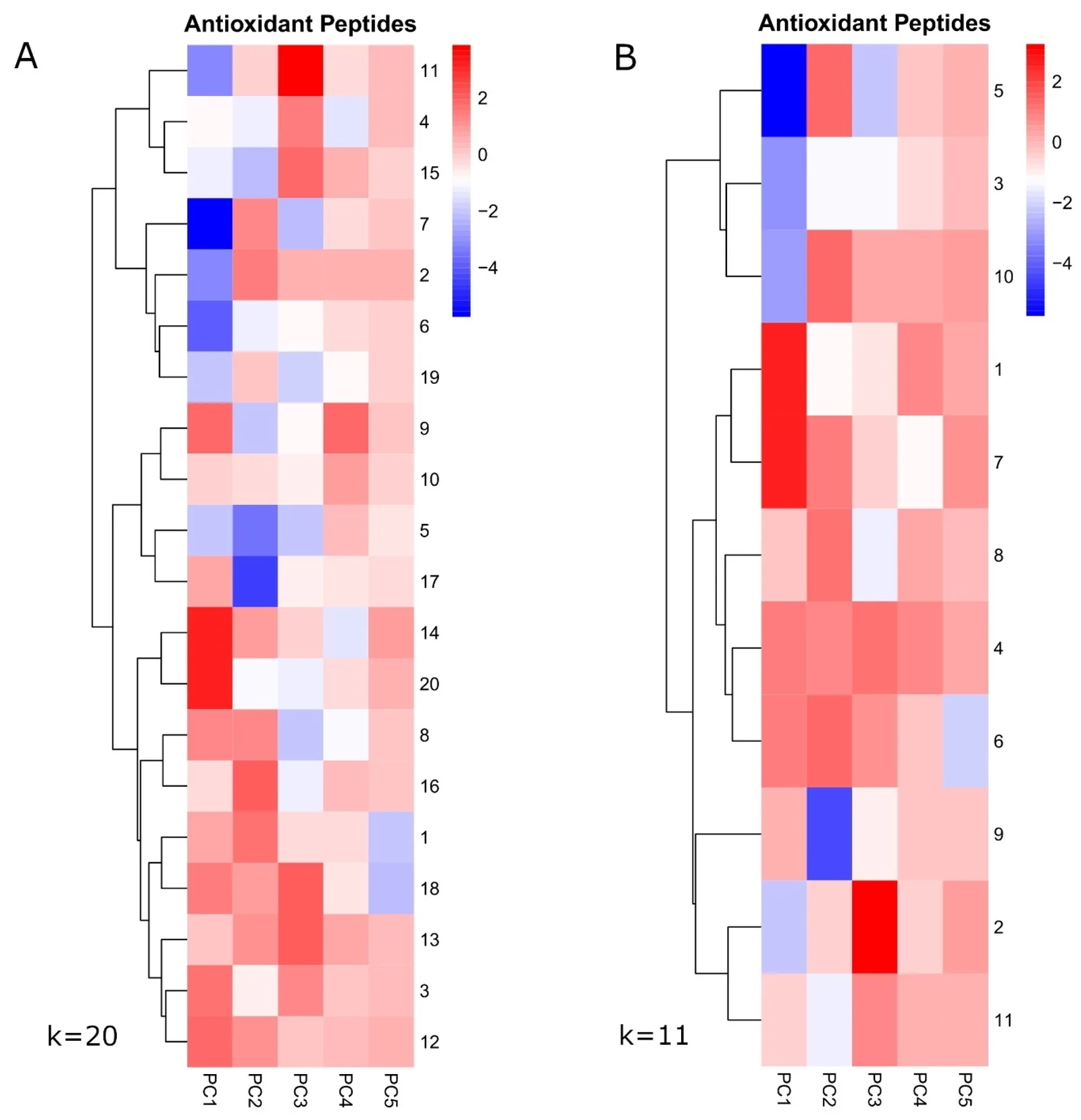
K-means clustering for the 620 peptides with the first five principal components. (**A**). Kmeans with k = 20, (**B**). Kmeans with k = 11. Analysis was made using pheatmap in RStudio.

The two k-means solutions provided complementary representations of the physicochemical organization of the peptide dataset. The k = 20 solution produced a finer partition, whereas k = 11 consolidated peptides into broader groups. Several collagen-derived candidates were assigned to clusters that also contained experimentally evaluated reference peptides in one or both clustering solutions. However, this co-clustering indicates only similarity according to the selected physicochemical descriptors and should not be interpreted as confirmation of antioxidant activity. Candidate prioritization (Table 3) was therefore based on the combined evaluation of PCA position, descriptor profiles, cluster membership under both resolutions, sequence redundancy, and synthetic feasibility. Experimental assays were subsequently required to determine whether the selected candidates exhibited antioxidant activity. The identified descriptor profiles, including moderate-to-low hydrophobicity, near-neutral or slightly negative charge, and favorable Boman index values, have previously been associated with peptide solubility and interactions with reactive species [42,43]. Nevertheless, these associations are descriptive and do not demonstrate a specific antioxidant mechanism or biological activity. Descriptor values and PCA loadings are provided in Supplementary Table S3, and cluster distribution is shown in Figure 1.

The PCA showed that the first three components explained 74.3% of the total variance, with PC1 (35.7%) and PC2 (21.0%) capturing most of the information, indicating effective dimensionality reduction. PC1 was mainly associated with structural and hydrophobic features (Cruciani and FASGAI descriptors, hydrophobicity, and aliphatic index), which are key for peptide–radical interactions. PC2 reflected electrostatic properties, including charge, pI, and aliphatic index, which are relevant to electron-transfer and metal-chelation mechanisms. PC3 incorporated charge and conformational descriptors, suggesting a role in redox activity, while PC4 was linked to interaction potential (Boman index), possibly related to indirect antioxidant effects. PC5 was primarily defined by molecular weight, indicating a secondary contribution of peptide size. Overall, antioxidant peptides were mainly differentiated by hydrophobicity, structural properties, and charge-related features, consistent with known determinants of antioxidant activity [23,44].

### 3.2. Experimental Validation of Antioxidant Activity

Experimental validation was performed using three approaches, two concentrations (0.2 and 0.4 mM), and three peptides reported as antioxidants as references, with Vitamin C as the gold standard.

#### 3.2.1. DPPH Scavenging Assay

**Figure 2:**
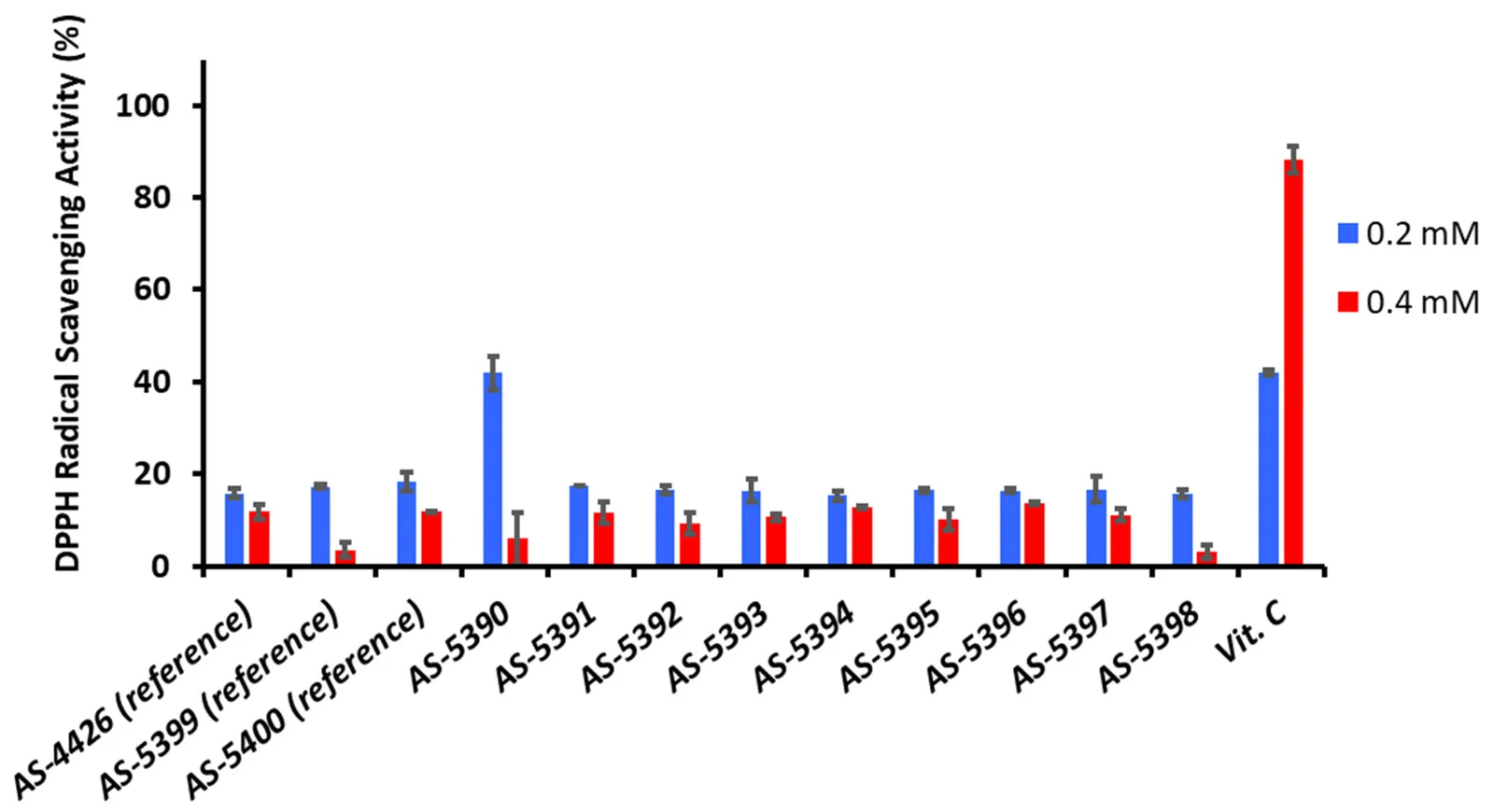
DPPH radical scavenging activity of the peptides at concentrations of 0.2 and 0.4 mM. Results are expressed as the mean ± standard deviation of three technical replicates. Significant global differences among peptide–concentration groups were detected using the Kruskal–Wallis test (H = 70.47, df = 25, *p* < 0.0001).

Figure 2 presents the results of the DPPH radical-scavenging assay at 0.2 and 0.4 mM. At the two preliminary screening concentrations evaluated, most peptides exhibited a non-monotonic concentration response, with greater apparent DPPH radical-scavenging activity at 0.2 mM than at 0.4 mM. Because only two concentrations were examined, this pattern should not be interpreted as a conventional concentration–response relationship. The Kruskal–Wallis test revealed significant global differences among the peptide–concentration groups in the DPPH assay (H = 70.47, df = 25, *p* < 0.0001). The mechanism underlying this response could not be established from the present data and is discussed together with the ABTS results in Section 3.2.2.

Among the predicted peptides (AS-5390-AS-5398), peptide AS-5390 stood out with the highest activity (41.85%), reaching values comparable to Vitamin C at the same concentration, indicating a strong electron-donating capacity. The remaining peptides exhibited moderate and relatively homogeneous activity (~15–18%), similar to the reference peptides (AS-4426, AS-5399, and AS-5400), suggesting a baseline antioxidant potential but lower efficiency than peptide AS-5390.

The high activity of peptide AS-5390 can be explained by its amino acid composition, particularly the presence of cysteine (C), whose thiol group (–SH) is widely recognized for its ability to donate electrons or hydrogen atoms, efficiently neutralizing free radicals. Furthermore, cysteine can form disulfide bonds, contributing to the structural stability of peptides and enhancing their redox behavior, as observed in antioxidant peptides rich in sulfur-containing residues [20,45].

#### 3.2.2. ABTS Assay

**Figure 3:**
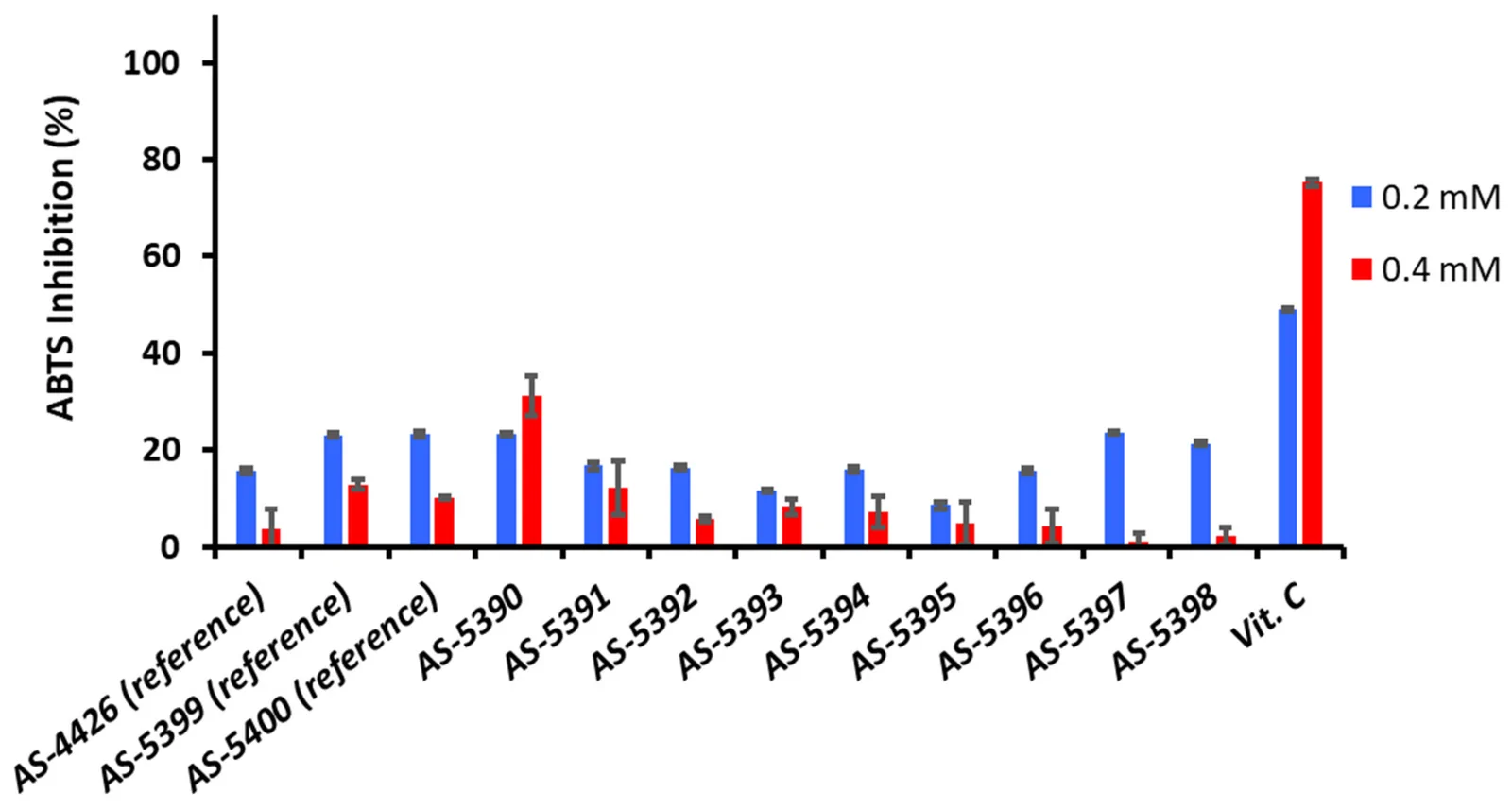
ABTS inhibition produced by the peptides at concentrations of 0.2 and 0.4 mM. Results are expressed as the mean ± standard deviation of three technical replicates. Significant global differences among peptide–concentration groups were detected using the Kruskal–Wallis test (H = 73.40, df = 25, *p* < 0.0001). No Trolox calibration was performed; therefore, results are expressed as percentage inhibition rather than TEAC.

The ABTS radical-scavenging results, expressed as percentage inhibition, are presented in Figure 3. This assay evaluates the ability of the peptides to inhibit the ABTS radical cation in an aqueous system and complements the DPPH assay because of its applicability to hydrophilic compounds and electron-transfer mechanisms [21]. No Trolox calibration was performed; therefore, the results are reported as ABTS inhibition (%) and not as TEAC values.

A different inhibition pattern was observed in the ABTS assay. At 0.2 mM, peptides AS-5390, AS-5397, and AS-5398, together with the reference peptides AS-4426, AS-5399, and AS-5400, exhibited the highest inhibition values, ranging from approximately 21% to 23%. At 0.4 mM, most peptides showed a marked decrease in ABTS inhibition, indicating lower radical-scavenging efficiency under these conditions.

The Kruskal–Wallis test showed significant global differences among the peptide–concentration groups in the ABTS assay (H = 73.40, df = 25, *p* < 0.0001). Taken together, the DPPH and ABTS results indicate a non-monotonic concentration response at the two preliminary screening levels evaluated, because most peptides displayed greater apparent radical-scavenging activity at 0.2 mM than at 0.4 mM. The occurrence of this general pattern in two different chemical assays indicates that it was not restricted to a single radical-scavenging method; however, it does not establish a common underlying mechanism.

Related concentration-, interaction-, and assay-dependent behaviors have been described for food-derived peptides. Díaz and Decker [46] reported that caseinophosphopeptides inhibited oxidation at concentrations below 1 mg/mL but became pro-oxidant above this concentration in a ferric/ascorbate-induced liposome model. Although this system differs from the DPPH and ABTS assays used here, it provides evidence that increasing peptide concentration does not necessarily result in greater antioxidant protection. Li et al. [47] demonstrated that particle characteristics, hydrophobicity, and the formation or reconstruction of intra- and intermolecular peptide interactions can differentially influence DPPH and ABTS antioxidant responses. Martineau-Côté et al. [48] also reported assay-dependent antioxidant performance and additive or synergistic interactions among individual food-derived peptides, although they did not observe an inverse concentration response.

Methodological factors may also contribute to the apparent ABTS response. Zheng et al. [49] demonstrated that amino acids and peptides can exhibit biphasic reaction kinetics in the ABTS assay and may fail to reach equilibrium during the commonly used incubation period of 6–10 min. Because the present assay used a 6 min endpoint, differences in reaction kinetics among peptide concentrations cannot be excluded. Nevertheless, peptide aggregation, turbidity, particle-size distribution, reaction kinetics, optical interference, and pro-oxidant markers were not directly evaluated in the present study. Therefore, self-association, reduced accessibility of redox-active residues, altered reaction kinetics, and changes in redox behavior should be regarded as plausible hypotheses rather than confirmed causes. A broader concentration range and direct physicochemical and kinetic measurements are required to determine the origin of this response.

Irrespective of the non-monotonic concentration response, the relative ABTS inhibition displayed by individual peptides may also be influenced by their amino acid composition. The observed differences among peptides may be associated with the presence of amino acid residues that favor solubility and conformational flexibility in aqueous media, facilitating interaction with the ABTS^+^ radical. In the case of AS-5390, glutamic acid and arginine may contribute to this behavior, as previously described for hydrophilic peptide systems [43]. In addition, acidic residues such as aspartic acid (D) and glutamic acid (E) contain carboxyl groups that may participate in electrostatic interactions and radical neutralization, thereby contributing to the observed ABTS inhibition [6,7,20,34,50].

Overall, the ABTS inhibition results indicate that several peptides exhibited moderate radical-scavenging capacity; multiple peptides possess moderate radical scavenging capacity, but none approach the high activity of Vitamin C, highlighting a limitation in their maximal antioxidant strength. However, it is essential to note that low activity in the ABTS assay does not necessarily imply a lack of overall antioxidant potential, as these peptides could exert their effect through alternative mechanisms, such as metal chelation or modulation of cellular antioxidant pathways, which are not detected by this specific chemical assay [51].

#### 3.2.3. Cellular Antioxidant Activity (CAA) Assay

**Figure 4:**
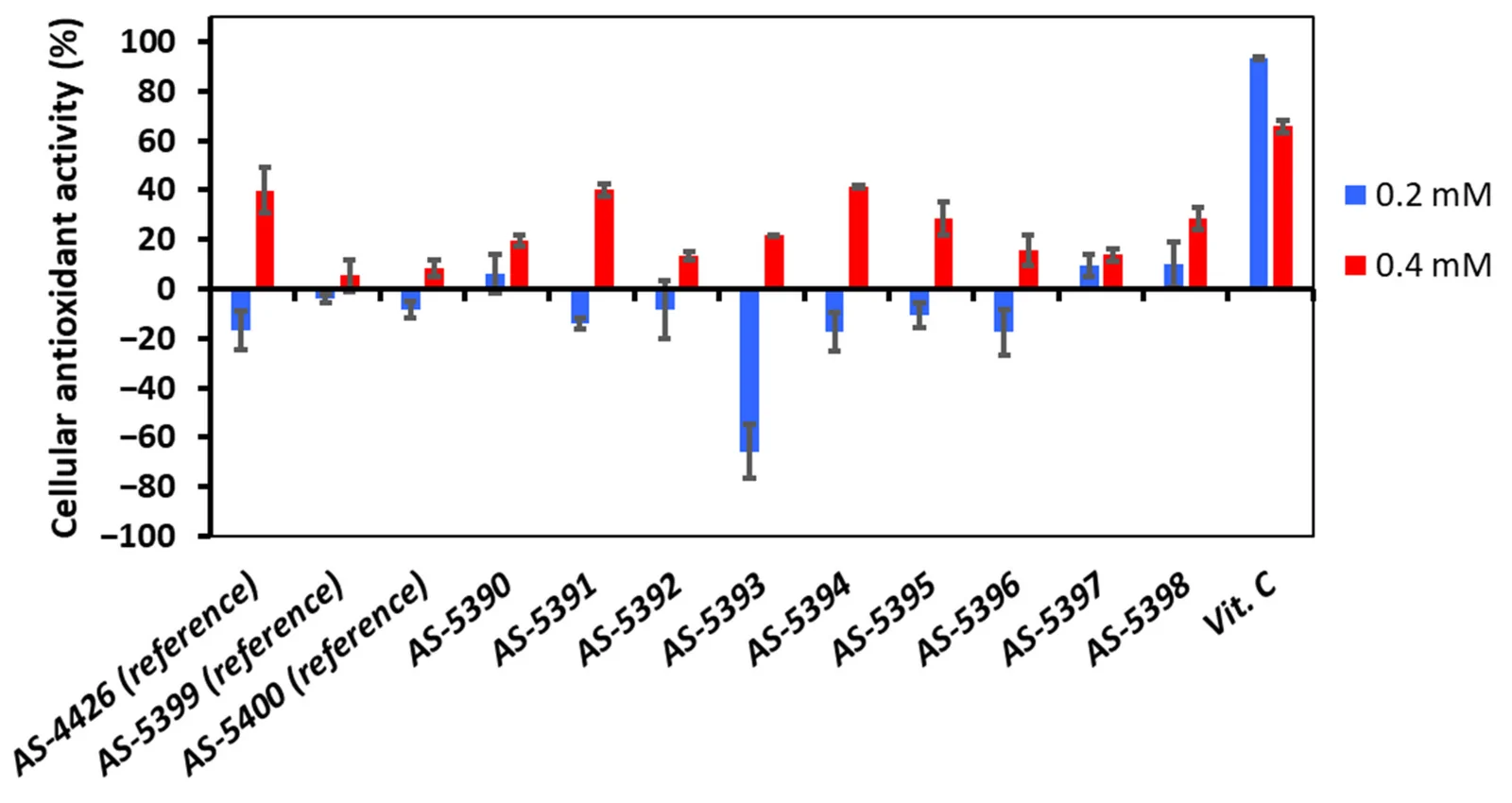
Cellular antioxidant activity of peptides at concentrations of 0.2 and 0.4 mM. Results are expressed as mean ± standard deviation of three technical replicates. Significant global differences among peptide–concentration groups were detected using the Kruskal–Wallis test (H = 50.15, df = 25, *p* = 0.0020).

Figure 4 presents the CAA of the synthesized peptides, evaluated in a live-cell model, which provides a more physiologically relevant approach to the antioxidant potential by integrating processes of cellular uptake, metabolism, and modulation of the intracellular redox balance.

The CAA assay revealed the most pronounced differences among peptides. At 0.2 mM, most peptides showed low or even negative values, suggesting limited intracellular antioxidant protection or potential pro-oxidant effects under these conditions. However, at 0.4 mM, several peptides exhibited a substantial increase in activity. Notably, peptides AS-5391, AS-5394, AS-5395, AS-5398 and AS-4426 reached the highest values (28–41%), indicating effective antioxidant activity in a cellular context. The Kruskal–Wallis test detected significant global differences among the peptide–concentration groups in the CAA assay (H = 50.15, df = 25, *p* = 0.0020). However, no individual pairwise comparison remained statistically significant after adjustment for multiple comparisons. Therefore, the higher responses observed for AS-5391, AS-5394, AS-5395, AS-5398, and AS-4426 were interpreted descriptively. This behavior suggests that, at low concentrations, these peptides do not effectively counteract ROS generation, or they may induce a mild pro-oxidant effect, a phenomenon previously described for certain antioxidant compounds in cellular systems [52], suggesting that higher concentrations are required for peptides to exert protective effects in more complex biological systems.

Peptide performance could be associated with the presence of small amino acids such as glycine (G), which confer structural flexibility to the peptide chain and may facilitate interaction with the cell membrane and subsequent internalization [23]. Furthermore, hydrophobic residues such as proline (P) and isoleucine (I) can increase the residence time of the peptide at the water–lipid interface, facilitating access to radicals in the lipid phase and helping to slow lipid peroxidation. Aspartic acid (D), for its part, provides a carboxylate group that can participate in the chelation of pro-oxidant transition metals (Fe^2+^/Cu^2+^), reducing reactions that generate reactive species [50].

This phenomenon is consistent with the complex nature of the CAA assay, where antioxidants can interact with cellular metabolic pathways, alter redox homeostasis, or even promote the secondary generation of ROS under certain conditions [52]. This result is particularly relevant, as the CAA assay reflects not only the chemical radical neutralization capacity of the peptide but also its biological effectiveness within the cell [7]. It is interesting that vitamin C shows greater antioxidant activity at lower concentrations, suggesting that higher doses do not necessarily translate into a greater protective effect at the cellular level. This behavior has been previously reported and is associated with saturation of cellular transporters and a possible redox imbalance induced by high concentrations of antioxidants [53,54].

Overall, the three assays highlight that antioxidant activity is highly dependent on both the mechanism and experimental conditions. The DPPH assay identified peptide AS-5390 as the most potent radical scavenger in a chemical system, whereas the ABTS assay showed a broader group of moderately active peptides, with little discrimination. In contrast, the CAA assay revealed that peptide AS-5394 is the most effective under biologically relevant conditions, outperforming even some reference peptides. In contrast, the CAA assay showed that AS-5394 exhibited the highest observed cellular antioxidant response. However, because no individual pairwise comparison remained significant after adjustment for multiple comparisons, this result should be interpreted descriptively and not as evidence of statistical superiority over the other peptides or reference sequences.

The different behavior observed between chemical assays and the CAA model suggests that their direct radical-scavenging capacity in solution does not solely determine the antioxidant performance of these peptides, but also by their ability to interact with the cellular environment. In this sense, AS-5390, which contains cysteine residues, may be particularly effective in chemical assays such as DPPH because thiol groups can directly participate in electron or hydrogen donation [20,45,50]. In contrast, the comparatively high cellular response observed for AS-5394 may be associated with mechanisms that depend on peptide–cell interactions rather than solely on direct radical neutralization. Its short sequence, PGDIG, combines glycine residues that provide conformational flexibility, proline and isoleucine residues that may favor interaction with the lipid–water interface of the cell membrane, and aspartic acid, whose carboxylate group can contribute to metal chelation and modulation of ROS-generating reactions [6,20,50].

Therefore, AS-5394 could exert its cellular antioxidant effect through a combination of improved membrane association, intracellular availability, metal-chelating capacity, and indirect regulation of oxidative processes. This interpretation supports the idea that cellular antioxidant activity depends on multiple mechanisms, including peptide uptake, intracellular localization, redox modulation, and interaction with pro-oxidant species, which are not fully captured by chemical assays such as DPPH or ABTS [7,20,23].

### 3.3. In Vitro Cytotoxicity Using WST-1 Assay

To assess whether the most active antioxidant candidates affected cell viability under the tested conditions, peptides AS-5390 and AS-5394 were evaluated using the WST-1 cell proliferation assay at 0.4 and 0.8 mM. Results were compared with untreated cells as the negative control and Triton X-100 as the positive cytotoxicity control.

**Figure 5:**
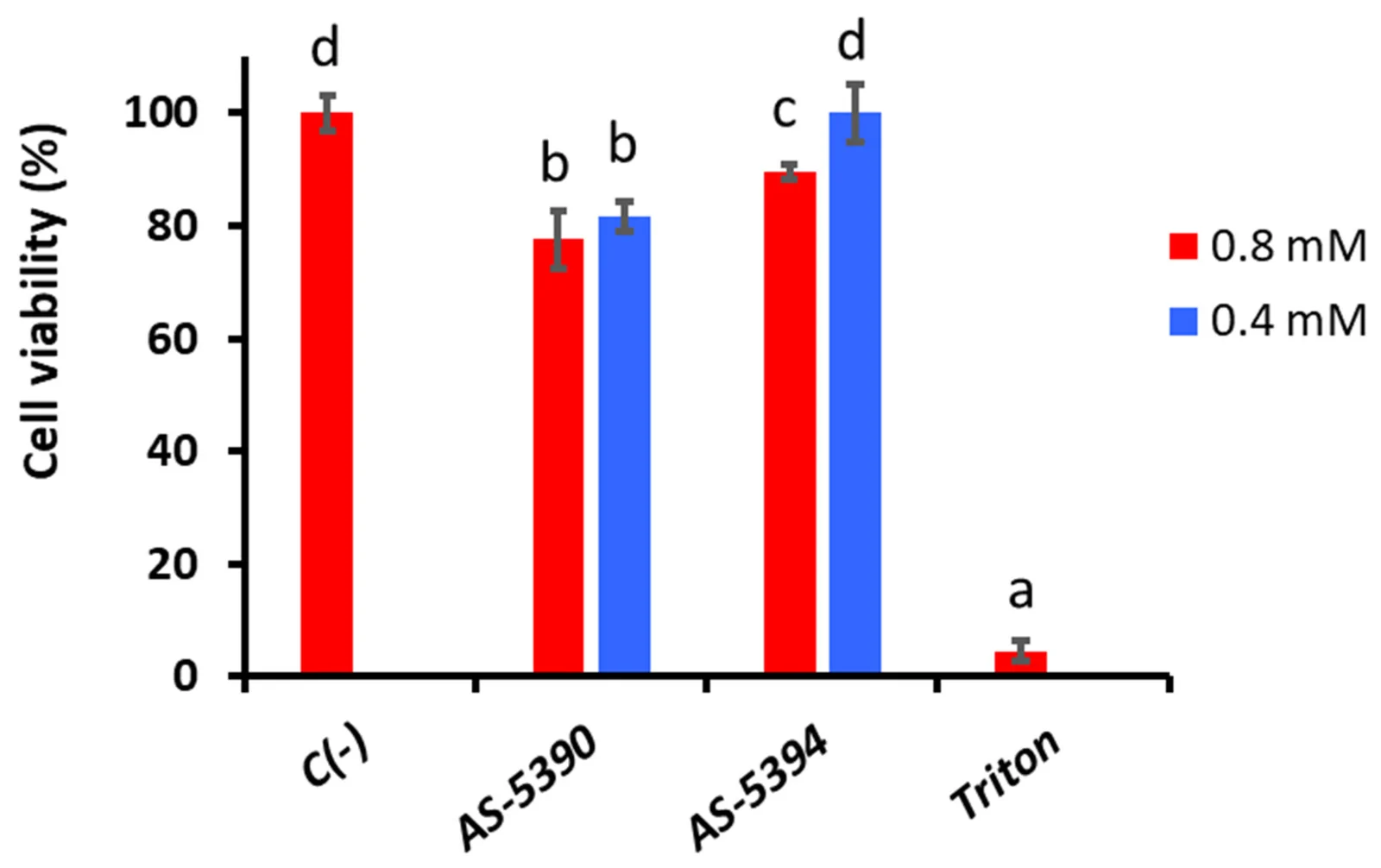
Cell viability of the RTgill-W1 salmonid cell line, assessed by the WST-1 cell proliferation assay after exposure to two concentrations (0.4 and 0.8 mM) of peptides AS-5390 and AS-5394. Untreated cells were used as the negative control (C−), while Triton served as the positive cytotoxicity control. Data are expressed as the mean ± standard deviation of three technical replicates. Different letters indicate statistically significant differences among groups (*p* ≤ 0.05).

As shown in Figure 5, both peptides maintained relatively high RTgill-W1 cell viability, with values exceeding 75% for AS-5390 and 90% for AS-5394. Notably, AS-5394 at 0.4 mM showed no statistically significant difference compared with the untreated-cell control. Within the evaluated concentration range, no clear concentration-dependent reduction in WST-1 metabolic activity was observed.

The Triton X-100 control produced a marked reduction in cell viability, confirming the responsiveness of the assay to cytotoxic conditions. These results indicate that AS-5390 and AS-5394 did not cause a pronounced reduction in RTgill-W1 metabolic activity or cell viability under the specific concentrations and exposure time evaluated. However, these findings should not be interpreted as evidence of general biocompatibility or safety, because only one cell line, two concentrations, and a single metabolic-activity assay were examined. Additional cell models, concentration ranges, exposure periods, and complementary cytotoxicity endpoints are required before broader safety conclusions can be established. It should also be noted that the WST-1 assay and the chemical antioxidant assays address different experimental endpoints. WST-1 reflects RTgill-W1 metabolic activity after exposure to AS-5390 and AS-5394 at 0.4 and 0.8 mM, whereas DPPH and ABTS evaluate direct radical-scavenging reactions in cell-free systems for the complete peptide set at 0.2 and 0.4 mM. Consequently, maintenance of cell viability does not demonstrate a monotonic antioxidant concentration response or exclude physicochemical and assay-dependent effects at the higher concentration.

Although the present study provides sequence-level evidence of antioxidant activity and preliminary cell-viability data, several limitations should be considered when interpreting these findings and translating them to real food-processing conditions. First, the peptides were evaluated as chemically synthesized and purified sequences in simplified in vitro chemical and cellular assays; therefore, their activity may differ when they are generated as part of complex salmon collagen hydrolysates, where peptide–peptide interactions, residual salts, lipids, proteins, and other matrix components can modify antioxidant performance. Furthermore, the WST-1 evaluation was restricted to the RTgill-W1 salmonid cell line, two peptide concentrations (0.4 and 0.8 mM), and a 48 h exposure period. Because WST-1 primarily reflects cellular metabolic activity, these results cannot establish general safety or biocompatibility. Future studies should include additional relevant cell lines, broader concentration ranges, longer exposure periods, and complementary endpoints such as membrane integrity, apoptosis, and oxidative damage.

Second, the in silico hydrolysis was performed using only subtilisin. Since each protease exhibits a specific cleavage pattern, the composition of the predicted peptide library is enzyme-dependent. Consequently, the peptide sequences generated in this study represent only those predicted under subtilisin hydrolysis and may differ from those obtained using other industrial or gastrointestinal proteases. Future studies should compare subtilisin with additional industrial proteases and gastrointestinal enzymes, such as pepsin, trypsin, and chymotrypsin, to determine whether alternative hydrolysis strategies generate additional antioxidant peptide candidates. In addition, the predefined minimum-length criterion excluded dipeptides, tripeptides, and tetrapeptides from the computational library. Because short peptides may also exhibit antioxidant activity, future studies should evaluate these excluded sequences using analytical strategies specifically designed for short-peptide prioritization and validation.

Third, food-processing operations such as heating, drying, freezing, pH adjustment, salt addition, storage, and exposure to oxygen or light may affect peptide stability, solubility, aggregation behavior, and radical-scavenging efficiency. In addition, only two preliminary screening concentrations were evaluated in the present study, which is insufficient to establish complete concentration–response profiles. Although most peptides exhibited lower apparent DPPH and ABTS activity at 0.4 mM than at 0.2 mM, the mechanism responsible for this non-monotonic response was not directly investigated.

Future studies should therefore evaluate a broader concentration range and incorporate peptide-specific optical blanks, time-resolved monitoring of radical-scavenging reactions, and direct measurements of solubility, turbidity, aggregation, and particle-size distribution. Assessing oxidation or pro-oxidant markers would also help determine whether the observed decrease reflects self-association, reduced accessibility of reactive residues, assay interference, altered reaction kinetics, or a genuine change in peptide redox behavior. Until these measurements are performed, these mechanisms should be considered plausible hypotheses rather than confirmed explanations. Accordingly, further studies involving turbidity, solubility, or particle-size distribution measurements will be required to determine whether the decrease in antioxidant activity observed at higher peptide concentrations is effectively associated with peptide aggregation or with other physicochemical phenomena that may reduce peptide availability and, consequently, antioxidant capacity. These analyses will help clarify the mechanism underlying the observed behavior and strengthen the interpretation of the results.

Fourth, the tested concentrations were selected for screening purposes and do not yet define the dose required to exert measurable antioxidant protection in actual food systems or during shelf-life studies. Accordingly, future work should validate AS-5390 and AS-5394 in real food matrices, particularly salmon or fish-based products, assessing oxidative stability, sensory impact, processing compatibility, digestive stability, regulatory feasibility, and cost-effective production from collagen by-products before industrial application can be proposed.

## 4. Conclusions

This study demonstrates that an integrated bioinformatic–experimental workflow can support the prioritization and validation of antioxidant peptide candidates derived from salmon collagen. Among the synthesized sequences, AS-5390 showed the strongest DPPH radical-scavenging activity, with performance comparable to vitamin C at 0.2 mM, whereas AS-5394 exhibited the highest cellular antioxidant activity, reaching approximately 40% at 0.4 mM. Both peptides maintained relatively high RTgill-W1 cell viability under the evaluated conditions.

These findings identify salmon collagen as a promising source of sequence-defined antioxidant peptides and support the potential valorization of salmon-processing by-products. However, the peptides were evaluated as synthetic sequences, the predicted library was generated using only subtilisin, and cellular viability was assessed using one cell line and a limited concentration range. Future studies should confirm peptide release in experimentally produced collagen hydrolysates and evaluate stability, activity, and cell responses in additional biological models and real food matrices under processing, storage, and digestion conditions.

## Figures and Tables

**Table 1:** Selected reference panel of experimentally evaluated antioxidant peptide sequences used for physicochemical comparison and candidate prioritization.

Amino Acid Sequence	Source	Method of Determination	Reference
VIAPW	Miiuy croaker muscle	DPPH, OH, O_2_, ORAC, CAA	[24,25]
VAAGRTDAGVH	Fermented fish sauce	DPPH	[26]
TSSSLNMAVRGGLTR	Ragi	DPPH	[27]
SAGNPN	Dry ham	DPPH	[28]
AWEEREQGSR	Amaranth	ORAC	[29]
YLAGKPQQEH	Amaranth	ORAC	[29]
IYIEQGNGITGM	Amaranth	ORAC	[29]
TEVWDSNEQ	Amaranth	ORAC	[29]
YSNQNGRF	Cottonseed	DPPH	[14]
GFPGRLDHWCASE	Flaxseed	ORAC	[30]
VVGGDGDV	Palm oil	DPPH	[31]
VPVTST	Palm oil	DPPH	[31]
LTTLDSE	Palm oil	DPPH	[31]
STTVGLGISMRSASVR	Ragi	DPPH	[27]
ALSTWTLQLGSTSFSASPM	Mackerel muscle	DPPH	[32]
LGTLLFIAIPI	Mackerel muscle	DPPH	[32]
YGRDEISV	Hemp seed	DPPH	[33]
LDLVKPQ	Hemp seed	DPPH	[33]
LDDPVFIH	Fermented fish sauce	DPPH	[26]
DHTKE	Egg white	DPPH	[34]
FFGFN	Egg white	DPPH	[34]
MPDAHL	Egg white	DPPH	[34]
SYPTECRMR	Sesame	DPPH	[35]

**Table 2:** *Salmo salar* collagen protein sequences retrieved from NCBI RefSeq and used as substrates for in silico enzymatic hydrolysis.

Name	Type	NCBI RefSeq Protein Accession Number	Length
Collagen, type I, alpha 1a [*Salmo salar*]	I	XP_014059932.1	1449
Collagen, type II, alpha 1b isoform X1 [*Salmo salar*]	II	XP_045547905.1	1490
Collagen alpha-1(IV) chain isoform X1 [*Salmo salar*]	IV	XP_045563445.1	1646
Collagen, type V, alpha 2a [*Salmo salar*]	V	XP_014019899.2	1495
Collagen alpha-3(VI) chain [*Salmo salar*]	VI	XP_045563874.1	1074
Collagen, type XI, alpha 1a isoform X1 [*Salmo salar*]	XI	XP_014012750.1	1820
Collagen alpha-1(XVI) chain-like isoform X2 [*Salmo salar*]	XVI	XP_045565559.1	1604
Collagen alpha-1(XXIV) chain-like [*Salmo salar*]	XXIV	XP_014024329.2	1719
Collagen, type XXVIII, alpha 1a [*Salmo salar*]	XXVIII	XP_014033389.2	1173

**Table 3:** In silico-predicted peptide candidates derived from *Salmo salar* collagen sequences and prioritized for chemical synthesis through exploratory PCA, physicochemical descriptor analysis, k-means clustering, sequence redundancy, and synthetic-feasibility criteria.

Peptide ID	Sequence	# Residues	Molecular Weight	Cluster Member	
				k = 20	k = 11
AS-5390	DGCERCF	7	829.01	9	2
AS-5391	GPEGKGL	7	656.83	12	4
AS-5392	GPQGF	5	504.61	3	7
AS-5393	GTPGS	5	417.48	12	7
AS-5394	PGDIG	5	457.55	3	1
AS-5395	VGEQGL	6	601.74	12	4
AS-5396	AGAPGES	7	587.67	9	11
AS-5397	VIQPL	5	568.78	17	9
AS-5398	VEENRDR	7	917.03	11	2
AS-4426	VIAPW (reference)	5	584.78	17	9
AS-5399	VAAGRTDAGVH (reference)	11	1053.29	10	8
AS-5400	TSSSLNMAVRGGLTR (reference)	15	1549.96	19	8

## Data Availability

The original contributions presented in this study are included in the article/Supplementary Materials. Further inquiries can be directed to the corresponding authors.

## Supplementary Materials

The following supporting information can be downloaded at https://www.mdpi.com/article/10.3390/foods15162882/s1: Table S1: Physicochemical descriptors from the Peptide package (Osorio et al., 2015) used in the clustering analysis [38]; Table S2: Peptides obtained from in silico hydrolysis of salmon collagen proteins with BIOPEP platform; Table S3: Principal component analysis (PCA) of the physicochemical descriptors. The table shows the proportion of explained variance for the first five principal components and the corresponding loading coefficients of the original descriptors. These loadings indicate the relative contribution of each descriptor to the principal components, which represent linear combinations of the original variables rather than individual physicochemical properties.
