# Supplementary material for: Bioinformatic Identification and Experimental Validation of Novel Antioxidant Peptides Derived from Salmon Collagen
Source: Foods. 2026 Aug 18;15(16):2882. doi: 10.3390/foods15162882 (PMC13511857; doi:10.3390/foods15162882)
Supplement: Supplementary file 1 [file foods-15-02882-s001.zip › foods-4466506-supplementary.pdf]

# Bioinformatic Identification and Experimental Validation of Novel Antioxidant Peptides Derived from Salmon Collagen

**Suleivys M. Nuñez [1,*], Camila B. Barrera [1], Ignacio S. del Río [1], Jaime E. Gómez López [1], Tanya Roman [2], Fanny Guzmán [3] and Constanza Cárdenas [3,4,*]**

[1] Escuela de Ingeniería Química, Pontificia Universidad Católica de Valparaíso, Valparaíso 2340025, Chile; camila.barrera.m@mail.pucv.cl (C.B.B.); ignacio.delrio.p@mail.pucv.cl (I.S.d.R.); jaime.gomez@pucv.cl (J.E.G.L.)
[2] Programa de Doctorado en Biotecnología, Pontificia Universidad Católica de Valparaíso/Universidad Técnica Federico Santa María, Valparaíso 2340025, Chile; tanya.roman.b@mail.pucv.cl
[3] Núcleo de Biotecnología Curauma (NBC), Pontificia Universidad Católica de Valparaíso, Valparaíso 2373223, Chile; fanny.guzman@pucv.cl
[4] Centro de Investigación Interdisciplinaria en Biomedicina, Biotecnología y Bienestar (CID3B), Pontificia Universidad Católica de Valparaíso, Valparaíso 2340000, Chile
* Correspondence: suleivys.nunez@pucv.cl (S.M.N.); constanza.cardenas@pucv.cl (C.C.); Tel.: +56-9-5320-9063 (S.M.N.)

## Supplementary material

**Table S1.** Physicochemical descriptors from the Peptide package (Osorio et al., 2015) used in the clustering analysis [38].

| Descriptor | Definition |
|---|---|
| aIndex | The aindex is defined as the relative volume occupied by aliphatic side chains. |
| Boman | This function computes the potential protein interaction index proposed by Boman. |
| Charge | This function computes the net charge of a protein sequence based on the Henderson-Hasselbalch equation described by Moore, D. S. |
| PP1 | Cruiciani descriptor related with Polarity. |
| PP2 | Cruiciani descriptor related with  Hydrophobicity. |
| PP3 | Cruiciani descriptor related with H-bonding. |
| F1 | Fasgai descriptor related with Hydrophobicity index. |
| F2 | Fasgai descriptor related with Alpha and turn propensities. |
| F3 | Fasgai descriptor related with Bulky properties. |
| F4 | Fasgai descriptor related with Compositional characteristic index. |
| F5 | Fasgai descriptor related with  Local flexibility. |
| F6 | Fasgai descriptor related with  Electronic properties. |
| Hidrophobicity | This function calculates the GRAVY hydrophobicity index of an amino acids sequence using Kidera scale. |
| pI | The isoelectric point (pI), is the pH at which a particular molecule or surface carries no net electrical charge. |
| MW | This function calculates the molecular weight of a protein sequence. |

**Table S3.** Principal component analysis (PCA) of the physicochemical descriptors. The table shows the pro-portion of explained variance for the first five principal components and the corresponding loading coefficients of the original descriptors. These loadings indicate the relative contribution of each descriptor to the principal components, which represent linear combinations of the original variables rather than individual physicochemical properties.

| | PC1 | PC2 | PC3 | PC4 | PC5 |
|---|---|---|---|---|---|
| **Proportion of Variance** | **35.7%** | **21.0%** | **18.2%** | **8.8%** | **6.1%** |
| **Cumulative Proportion** | **35.7%** | **56.1%** | **74.3%** | **83.1%** | **89.2%** |

| Original Descrtiptors | PC1 | PC2 | PC3 | PC4 | PC5 |
|---|---|---|---|---|---|
| **aIndex** | -0.0073 | -0.4641 | -0.1542 | 0.2380 | -0.1412 |
| **Boman** | -0.3460 | 0.2479 | 0.1871 | -0.0754 | 0.0384 |
| **Charge** | -0.1432 | 0.1820 | -0.5125 | -0.0218 | 0.0362 |
| **PP1** | -0.3718 | 0.0845 | 0.2649 | 0.0250 | 0.0507 |
| **PP2** | -0.3201 | -0.2324 | -0.2043 | -0.2855 | -0.0680 |
| **PP3** | -0.0183 | 0.3695 | -0.3987 | 0.1740 | 0.0357 |
| **F1** | 0.2003 | -0.3465 | -0.2356 | -0.0504 | -0.0388 |
| **F2** | -0.3053 | -0.3143 | 0.0805 | 0.2162 | -0.0809 |
| **F3** | -0.2979 | -0.3663 | -0.0833 | -0.1502 | -0.0801 |
| **F4** | 0.2455 | 0.0143 | -0.1580 | 0.5524 | -0.1082 |
| **F5** | 0.2011 | 0.0277 | -0.0697 | -0.6115 | 0.0997 |
| **F6** | -0.3697 | -0.0999 | -0.2287 | 0.0433 | -0.0275 |
| **Hidrophobicity** | -0.3562 | 0.2285 | 0.1541 | 0.2164 | -0.0233 |
| **pI** | -0.1728 | 0.2130 | -0.4763 | -0.0723 | -0.0601 |
| **MW** | 0.0498 | 0.1754 | 0.0771 | -0.1417 | -0.9632 |
